# Exploring Adiposity and Chronic Kidney Disease: Clinical Implications, Management Strategies, Prognostic Considerations

**DOI:** 10.3390/medicina60101668

**Published:** 2024-10-11

**Authors:** Lasin Ozbek, Sama Mahmoud Abdel-Rahman, Selen Unlu, Mustafa Guldan, Sidar Copur, Alexandru Burlacu, Adrian Covic, Mehmet Kanbay

**Affiliations:** 1 Department of Medicine, Koç University School of Medicine, Istanbul 34450, Turkey; lozbek18@ku.edu.tr (L.O.); srahman20@ku.edu.tr (S.M.A.-R.); sunlu20@ku.edu.tr (S.U.); mguldan17@ku.edu.tr (M.G.); 2 Department of Internal Medicine, Koç University School of Medicine, Istanbul 34450, Turkey; scopur14@ku.edu.tr; 3 Faculty of Medicine, University of Medicine and Pharmacy “Grigore T Popa”, 700115 Iasi, Romania; adrian.covic@umfiasi.ro; 4 Institute of Cardiovascular Diseases “Prof. Dr. George I.M. Georgescu”, 700503 Iasi, Romania; 5 Nephrology Clinic, Dialysis, and Renal Transplant Center “C.I. Parhon” University Hospital, 700503 Iasi, Romania; 6 Department of Medicine, Division of Nephrology, Koç University School of Medicine, Istanbul 34450, Turkey

**Keywords:** obesity, sarcopenic obesity, visceral adiposity, kidney function, glomerular filtration rate

## Abstract

Obesity poses a significant and growing risk factor for chronic kidney disease (CKD), requiring comprehensive evaluation and management strategies. This review explores the intricate relationship between obesity and CKD, emphasizing the diverse phenotypes of obesity, including sarcopenic obesity and metabolically healthy versus unhealthy obesity, and their differential impact on kidney function. We discuss the epidemiological evidence linking elevated body mass index (BMI) with CKD risk while also addressing the paradoxical survival benefits observed in obese CKD patients. Various measures of obesity, such as BMI, waist circumference, and visceral fat assessment, are evaluated in the context of CKD progression and outcomes. Mechanistic insights into how obesity promotes renal dysfunction through lipid metabolism, inflammation, and altered renal hemodynamics are elucidated, underscoring the role of adipokines and the renin–angiotensin–aldosterone system. Furthermore, the review examines current strategies for assessing kidney function in obese individuals, including the strengths and limitations of filtration markers and predictive equations. The management of obesity and associated comorbidities like arterial hypertension, type 2 diabetes mellitus, and non-alcoholic fatty liver disease in CKD patients is discussed. Finally, gaps in the current literature and future research directions aimed at optimizing the management of obesity-related CKD are highlighted, emphasizing the need for personalized therapeutic approaches to mitigate the growing burden of this intertwined epidemic.

## 1. Introduction

The increasing prevalence of obesity among chronic kidney disease (CKD) patients underscores the critical need for targeted research into various obesity types and their implications for personalized treatment strategies within this population [1,2]. Obesity significantly increases the risk of CKD, with obese individuals having a 1.83 times higher risk (95% CI 1.57–2.13), and obesity is estimated to account for 24.2% of CKD cases in men and 33.9% in women [3]. Concurrently, the rising incidence of diabetes mellitus (DM), arterial hypertension (AH), and obesity, alongside the prevalence of CKD, requires urgent evaluation to mitigate the far-reaching impact of the cardiovascular–kidney–metabolic syndrome on global health systems [4]. This impact is evidenced by escalating rates of all-cause mortality and disease burden [5,6,7,8].

Obesity in CKD patients varies significantly in fat distribution and metabolic phenotype, impacting disease progression and treatment outcomes. Sarcopenic obesity, marked by muscle wasting and excess adiposity, complicates CKD management by worsening metabolic control and increasing frailty risk, requiring strategies to preserve muscle mass and manage obesity-related comorbidities [9]. Additionally, distinguishing between metabolically healthy and unhealthy obesity is crucial in predicting the prognosis of CKD patients [10]. However, despite these challenges, there remains a noticeable absence of comprehensive assessments regarding fat distribution, metabolic phenotypes of obesity, and conditions such as sarcopenic obesity in guiding personalized clinical management approaches for CKD patients. For CKD patients with obesity, a comprehensive approach is crucial, integrating pharmacological interventions, lifestyle modifications, and, when indicated, endoscopic and surgical options [11]. Beyond adiposity evaluation, a global CKD management approach must address diverse obesity phenotypes and specific fat distributions, ensuring personalized strategies that effectively manage both CKD progression and obesity-related comorbidities [12,13].

This review aims to explore the multifaceted interplay between obesity and CKD, focusing on how obesity contributes to the development and progression of CKD through mechanisms such as lipid metabolism, inflammation, and renal hemodynamics, while also complicating CKD management due to challenges in accurately assessing kidney function. It highlights the importance of considering different obesity phenotypes, such as sarcopenic obesity and metabolically healthy obesity (MHO), and discusses emerging therapeutic strategies, including dietary interventions, exercise, and pharmacological treatments like glucagon-like peptide (GLP-1) and glucose-dependent insulinotropic polypeptide (GIP) receptor agonists.

## 2. Body Weight Dynamics and Body Composition: Impact on CKD Risk and Mortality

Elevated body mass index (BMI) or obesity was individually shown to be associated with an increased risk of developing CKD, although the relationship can vary in strength across different clinical studies and systematic reviews [14,15]. In this context, a large-scale prospective cohort study involving 1,405,016 adults aged 20–79 found that higher BMI levels, starting from overweight (BMI 25 to <30 kg/m^2^) and increasing to obesity (BMI ≥ 30 kg/m^2^), were consistently associated with progressively increased risks of advanced CKD stages 4–5, with a particularly pronounced risk at a BMI ≥ 35 kg/m^2^ [16]. At this point, the debate continues regarding whether excess adipose tissue alone, metabolic factors, or both collectively contribute to the increased risk of CKD [17], particularly in light of the emergence of a subgroup of patients classified as having MHO. MHO, despite excess adiposity, may present fewer metabolic complications compared to its unhealthy counterpart, influencing disease progression differently within the CKD context [18,19,20]. Moreover, intra-abdominal and perirenal fat distributions are linked to heightened cardiovascular risk and kidney dysfunction through mechanisms involving increased inflammation, adipokine release, local activation of the renin–angiotensin–aldosterone-system (RAAS), and subsequent vascular complications such as vascular calcification and atherosclerosis [21,22]. A large-scale British cohort study spanning 20 years and involving over 4.4 million adults free of CKD and cardiovascular disease at baseline found that individuals with metabolically healthy overweight had a 30% increased risk of developing CKD, while those with MHO faced a 66% higher risk, compared to those with normal weight and no metabolic abnormalities [23]. This association was particularly pronounced in individuals under 65 years old and showed a graded increase in risk with the presence of multiple metabolic abnormalities, highlighting the significant impact of excess weight, even in the absence of traditional metabolic disorders, on the development of CKD. However, conflicting findings exist in the literature [17]. Moreover, the dynamic nature of obesity and metabolic health adds further complexity to this issue. A study conducted by Cho et al. found that a significant proportion of individuals transitioned to different metabolic health and obesity categories during the study period, in addition to showing that individuals classified as MHO had a higher risk of CKD compared to those who were MHO and non-obese [24]. Individuals who transitioned from MHO to metabolically healthy and non-obese did not exhibit an increased risk of CKD, suggesting that maintaining or achieving metabolic health may mitigate the risk associated with obesity alone. Conversely, those who remained in the stable MHO category or transitioned to metabolically unhealthy states (either obese or non-obese) had a higher risk of developing CKD [24]. In a large cohort study of 176,420 Austrian adults followed for over two decades, it was demonstrated that approximately 42% of the association between BMI and end-stage kidney disease (ESKD) risk could be attributed to mediation through the triglyceride–glucose index, a marker of insulin resistance [25].

Excess body weight and obesity exert profound influences not only on the risk of CKD but also on progression through multifaceted mechanisms, including confounding factors such as glomerular hyperfiltration, RAAS activation, insulin resistance, type 2 DM, AH, endothelial dysfunction, lipotoxicity, and excess visceral fat, which further exacerbate chronic inflammation and insulin resistance, also compromising kidney health and adding to cardiovascular risks through concurrent dyslipidemia [26]. Nevertheless, clinical studies on obese individuals with CKD have shown better survival outcomes compared to their non-obese counterparts, highlighting the “obesity paradox” in this context [27]. A systematic review and meta-analysis, encompassing 484,906 participants from 10 studies, revealed that while being underweight was associated with higher mortality risk, being overweight and mild obesity (class I) were linked to a lower mortality risk in CKD stages 3–5 [27], suggesting an obesity paradox in the advanced CKD population. This paradox has also highlighted concerns about the limitations of BMI as a measure of body fat content, potentially leading to a perception of improved survival outcomes among obese patients with CKD [28]. A study conducted in a cohort of 453,946 US veterans with non-dialysis-dependent CKD found a U-shaped relationship between BMI and outcomes, with overweight and mildly obese patients (BMI 25 to <35 kg/m^2^) having the best prognosis. BMI levels below 25 kg/m^2^ were consistently associated with worse outcomes, independent of CKD severity, while BMI levels ≥ 35 kg/m^2^ showed worse outcomes in earlier CKD stages but not in advanced CKD (estimated glomerular filtration rate (eGFR) < 30 mL/min per 1.73 m^2^) [29], emphasizing that BMI and CKD prognosis have a nonlinear relationship and are affected widely by the stage of CKD.

The obesity paradox refers to the observation that individuals with obesity often have better survival outcomes in certain chronic diseases, such as heart failure and CKD, compared to those with normal weight [30]. Several mechanisms have been proposed to explain this phenomenon. One key hypothesis suggests that individuals with obesity have larger energy reserves in the form of adipose tissue and muscle mass, which may help them tolerate the metabolic stress associated with chronic illness, such as prolonged catabolism [31,32,33]. Additionally, individuals with MHO maintain favorable metabolic profiles with preserved insulin sensitivity, better lipid levels, and lower systemic inflammation, which could explain their reduced risk for adverse outcomes [34]. Methodological factors, such as selection bias, may also play a role, as patients with obesity are often diagnosed earlier and receive more intensive medical interventions [35]. Furthermore, frailty in chronic disease is associated with poor prognosis, and individuals with sarcopenic obesity may be more prone to early complications, reflecting a rapid disease progression [36,37]. Taken together, these factors highlight the complexity of interpreting BMI in the context of chronic disease outcomes.

While BMI is crucial for diagnosing obesity and assessing associated risks, variations in body fat distribution, metabolic health, and accompanying comorbidities and cardiovascular risks among individuals with similar BMI highlight the need to consider additional measures of adiposity in the CKD population [28].

## 3. Obesity Measures among CKD Patients

Obesity is linked with a variety of additional health conditions, including, but not limited to, components of metabolic syndrome, atherosclerotic cardiovascular disease, dyslipidemia, and insulin resistance [38]. Various measures of obesity include BMI, waist circumference (WC), waist-to-hip ratio (WHR), waist-to-height ratio (WHtR), skinfold thickness, body fat percentage, and visceral fat assessment, each providing unique insights into body composition and associated health risks [38]. It is crucial to understand which specific measures of obesity are most relevant in CKD patients, as accurate assessment can provide better estimates of cardiovascular risk, metabolic health status, stratification for complications, tailored treatment planning, and improved clinical outcomes.

Most studies on the impact of obesity in the CKD population utilizing BMI as a measurement of obesity found a negative relationship between an increase in BMI and the corresponding risk of a decrease in eGFR or albuminuria [39]. While BMI is simplistic, widely used, and became the first assessment tool in the diagnosis and classification of obesity, there is a significant number of BMI-related errors reported, including its oversight of variations in body composition such as muscle mass and bone density, a nonlinear relationship with body fat that results in individuals with similar BMIs having significantly different body fat percentages, and biased associations depending on the study design employed [40]. A cross-sectional study investigating the limitations of BMI in assessing obesity among patients with CKD compared to other methods such as air displacement plethysmography and skinfold thickness measurements found that BMI, while sensitive in identifying obesity (100% sensitivity), had poor specificity (72%) and a low negative predictive value (30%), often misclassifying individuals without obesity as obese [28]. The prevalence of obesity according to BMI was significantly lower compared to air displacement plethysmography, which showed higher obesity rates in both controls (60%) and CKD patients (90%). Skinfold thickness measurements demonstrated superior accuracy (94%) in classifying obesity among CKD patients compared to BMI and bioelectrical impedance analysis [28]. The study highlighted that BMI frequently missed cases of subclinical obesity, particularly in individuals with low lean body mass. Similarly, a study conducted on 730 elderly women, but this time to analyze the CKD risk, revealed that abdominal obesity, particularly defined by WHtR ≥ 0.6, WC, and WHR, was significantly associated with CKD stage ≥ 3 [41]. BMI also showed a notable correlation with CKD [41]. However, among the anthropometric measures assessed, WHtR emerged as the most predictive index for CKD risk in this population [41], with BMI and WHR following behind in receiver operating characteristic analysis.

It is noteworthy to indicate that prior studies discussed in this section consistently highlight variations in how obesity metrics and cut-offs for predicting the outcomes in CKD populations are significantly influenced by age, gender, race, and CKD stage. It is widely recognized that the distribution of body fat and the proportion of muscle mass vary significantly between women and men as well as among different races and age groups, leading to diverse metabolic and prognostic implications. A multicenter prospective cohort study found that among 2833 Korean maintenance HD patients, men with a BMI greater than 25.1 kg/m^2^ showed a significantly reduced mortality risk, whereas this association was not observed in women [42]. Additionally, increases in BMI over 12 months and higher serum creatinine levels were linked to improved survival outcomes, specifically in male patients [42]. A prospective cohort study in Sweden analyzed data from 26,723 subjects and found that higher BMI, WC, WHR, WHtR, and weight were associated with an increased risk of CKD in both genders [43]. Specifically, body fat percentage was significantly linked to CKD risk in women (HR 2.01, 95% CI: 1.45–2.78), whereas BMI (HR 1.51, 95% CI: 1.18–1.94) and weight (HR 1.52, 95% CI: 1.19–1.94) showed stronger associations in men [43]. These findings again highlight the gender-specific variations in obesity-related CKD risk within a large, longitudinal study population. At this juncture, it is also crucial to recognize the pivotal role of sex hormones in metabolic health. Estrogen’s protective effects in pre-menopausal patients underscore gender variations, while age notably influences pre- and post-menopausal women, emphasizing these distinctions [44]. Given these findings, it is crucial not only to assess the type of obesity measures and their corresponding thresholds among CKD patients but also to tailor these thresholds based on various demographic characteristics of the patients. Future studies should focus on developing prognosis prediction models that incorporate variables such as age, gender, ethnicity, CKD staging, comorbidities, and possibly menopausal status. This approach would enable the determination of individualized cut-off values rather than relying on a universal set of thresholds for all patients. Despite limited literature proposing alternative cut-off values for measuring obesity using both BMI and other measures, a study found that BMI thresholds of ≥25 kg/m^2^ are more accurate in identifying obesity in this population compared to conventional thresholds [45], highlighting the importance of detecting significant cut-off values for the evaluation of obesity in individuals with CKD.

## 4. The Implications of the Types of Obesity in CKD

**Figure 1:**
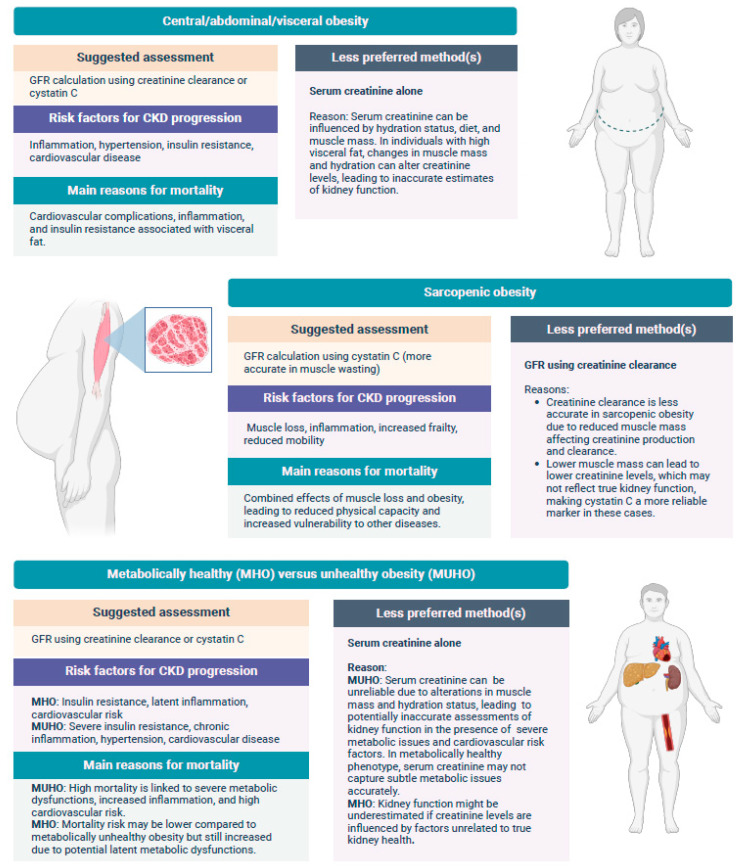
Methodologies to predict CKD progression and to assess renal function among various types of obesity.

The distribution of body fat and its impact on cardiovascular outcomes has been extensively studied [46] (Figure 1). However, understanding body fat distribution specifically in CKD patients requires further elucidation, given the limited number of studies exploring its impact on incidence as well as its prognostic implications. Nevertheless, emerging evidence on the prognostic significance of central, peripheral, visceral, and subcutaneous obesity among CKD patients continues to provide promising insights.

Additionally, the concept of sarcopenic obesity, a relatively new phenomenon, is beginning to be explored more deeply in CKD literature.

Central obesity refers to the accumulation of adipose tissue primarily around the abdomen and upper body, particularly around the waist, while peripheral obesity refers to the accumulation primarily around the hips, thighs, and buttocks, often depicted as a pear-shaped body appearance. WC, WHR, and hip circumference are the commonly employed methods of measurement for the evaluation [47]. WHtR, a measure of central obesity, was also found to be independently associated with CKD, even after adjusting for other components of metabolic syndrome. Moreover, the association between central obesity and CKD was strengthened in the presence of elevated plasma C-reactive protein (CRP) levels, indicating that inflammation modifies the impact of central obesity on CKD risk [48]. It was shown that higher baseline WC was independently associated with an increased risk of CKD development, even after adjusting for various confounding factors [49]. Specifically, each category’s increase in WC showed a trend toward higher CKD risk [49]. In contrast, WHR did not independently predict CKD incidence, and BMI showed less predictive power compared to WC [49]. Individuals categorized as having normal weight but with central obesity, determined by WHR, have notably elevated risks of both overall and cardiovascular mortality compared to those without central obesity but with a normal BMI [50].

Visceral obesity specifically means the buildup of adipose tissue around the internal organs, commonly the liver, pancreas, and intestines, while the adipose tissue in subcutaneous obesity deposits under the skin, which corresponds to areas including thighs, hips, and abdomen, where this phenomenon most likely occurs. The measurement of these two types is not as straightforward as other types, requiring radiological body imaging techniques such as MRI and CT, bioelectrical bioimpedance analysis, skinfold calipers, and body fat percentage calculation. Higher visceral adiposity index (VAI) levels were independently associated with an increased risk of developing CKD in both men and women, with progressively higher hazard ratios observed across quartiles of VAI [51]. The same study also demonstrated that VAI had predictive value for CKD incidence, particularly showing superior predictive performance in men compared to traditional risk factors [51]. Interestingly, a recent meta-analysis evaluated the diagnostic accuracy of the VAI for CKD, concluding that the VAI demonstrates moderate sensitivity (67%) and specificity (75%) in identifying CKD across a diverse population [52]. The findings suggest that the VAI could serve as a useful tool for predicting CKD, though additional research is needed to validate its diagnostic utility further. The impact of visceral adiposity on CKD extends beyond its role in incidence and diagnostics to include prognosis as well. CKD patients with higher visceral fat, defined by a visceral to subcutaneous fat ratio > 0.55, had a three-fold higher risk of experiencing cardiovascular events compared to those with lower visceral fat levels [53], highlighting the significant predictive value of visceral obesity for cardiovascular outcomes in this population. Higher visceral fat area, even below the threshold of 100 cm^2^, was independently associated with an increased risk of kidney disease progression, assessed by ≥50% eGFR decline or ESKD, after a follow-up of nearly 12 years [54], highlighting the visceral fat area as a versatile predictor of CKD prognosis across different disease stages and comorbid conditions like hyperglycemia and DM [54]. Further research is required to establish its prognostic value. The potential role of VAI as a predictor of CKD pathogenesis highlights its association with inflammation and metabolic dysregulation, as demonstrated by the fact that higher VAI scores were linked to decreased GFR and increased CKD risk, particularly in males and females with elevated levels of inflammatory markers such as highly sensitive CRP, interleukin-6 (IL-6), and other factors like systolic and diastolic blood pressure [55].

Sarcopenic obesity is defined by the coexistence of obesity and sarcopenia, characterized by low skeletal muscle mass and function [50]. It typically results from aging, sedentary behavior, metabolic issues linked to excess body fat, and chronic diseases like DM and heart disease [50]. Aging-related loss of skeletal muscle mass and function commonly leads to sarcopenic obesity, which can also manifest independently in individuals with obesity at any age, driven by metabolic changes, oxidative stress, inflammation, insulin resistance, altered muscle metabolism, and sedentary lifestyles [50]. Sarcopenic obesity increases the risk of frailty, disability, and mortality [50]. Diagnosis involves screening for high BMI or WC, coupled with assessments of muscle strength and function [50]. A study found that sarcopenic obesity significantly increased the risk of incident CKD, highlighting the specific role of combined muscle loss and obesity in CKD progression among type 2 DM patients with preserved kidney function [56]. On the other hand, another study investigated how different diagnostic criteria for sarcopenic obesity in CKD lead to varied prevalence estimates. Muscle impairment prevalence ranged from 11% to 50%, obesity from 26% to 62%, and sarcopenic obesity from 2% to 23%. The agreement between diagnostic methods was inconsistent, indicating the need for standardized criteria to accurately diagnose sarcopenic obesity and its components among CKD patients [57]. In CKD patients, excess adiposity appeared to mitigate the mortality risk associated with sarcopenia, suggesting a complex interaction between body composition and mortality outcomes that warrants consideration in future clinical interventions (4). A study investigating body composition differences across eGFR categories in adults from the NHANES 1999–2004 using dual-energy x-ray absorptiometry found a higher prevalence of sarcopenia and sarcopenic obesity in lower eGFR groups (P trend < 0.01 and <0.001, respectively). Sarcopenia was independently associated with stage 4 CKD among older adults (adjusted OR 2.58, 95% CI 1.02–6.51). Importantly, BMI underestimated obesity, particularly pronounced in eGFR 15–29 mL/min/1.73 m^2^ (71% obese by dual-energy X-ray absorptiometry vs. 41% by BMI) and was highly inaccurate in obese individuals with sarcopenia (97.7% misclassified) [58]. Using appropriate measures for obesity becomes more important in CKD patients to predict outcomes and manage appropriately, particularly in the setting of sarcopenic obesity in the CKD population, as they are theoretically at risk of developing sarcopenia. Beyond the mentioned classifications of obesity based on body fat distribution, location, and muscle mass, obesity is commonly characterized based on the presence or absence of metabolic or cardiovascular burden, which emerged with the detection of MHO subgroups [59,60,61,62]. Studies have shown that individuals classified as metabolically unhealthy obese also face a higher risk of developing incident CKD compared to metabolically healthy normal-weight counterparts [20,63]. Overall, despite the satisfactory evidence profile regarding the incidence of CKD in patients with obesity, there are currently limited studies on the prognostic aspect of CKD in the obese population, although the above evidence on central and visceral obesity overall might implicitly suggest that the prognosis of CKD in the setting of obesity might be more associated with the metabolic and cardiovascular complications. Similarly, visceral obesity has been associated with worse clinical and metabolic outcomes, such as the association between pancreatic steatosis and DM, or fatty kidney disease and CKD [64,65]. Future studies exploring if the MHO phenotype has a different prognostic CKD profile compared to individuals with metabolically healthy non-obesity can assist us in understanding more about this issue.

## 5. Measures for Assessing Kidney Function in CKD

Clinically, the most reliable methods for assessing kidney function are checking for albuminuria and measuring the GFR, described as the urinary or plasma clearance of certain endogenous or exogenous markers. Gold standard methods to obtain measured GFR (mGFR) with exogenous filtration markers such as iothalamate, EDTA, diethylene triamine pentaacetic acid, and iohexol are not used in clinical practice as they are expensive, difficult to implement, and time-consuming [66]. Therefore, we turn to eGFR using endogenous filtration markers, most notably creatinine and cystatin C (CysC) [67]. Mathematically estimating GFR using different formulas may prove to be a more accurate assessment of true GFR compared to 24 h urinary clearance of creatinine (ClCr) [68]. However, there are cases where 24 h urinary collection and measured ClCr are needed; for example, in those with low or high protein intake, those taking creatine supplementation or a diet rich in meat, those with muscle mass abnormalities, or those with rapid change in kidney function [69]. Other biomarkers, such as albuminuria, have a strong correlation with kidney function decline and outcomes and are used in conjunction with GFR to evaluate kidney function [70].

CKD is defined as a GFR < 60 mL/min per 1.73 m^2^ for a duration of ≥3 months, regardless of the underlying cause [67,71]. In accordance with the current clinical practice guidelines, eGFRcr should be the initial test when assessing GFR, and confirmatory tests including eGFR based on CysC (eGFRcys) or mGFR are mostly performed when greater accuracy is required [72,73]. Research has shown the efficacy of eGFRcr-cys measurements, particularly in investigations involving race, which is linked to variations in body size and composition, highlighting discrepancies in GFR estimates based on creatinine. As CysC has weaker associations with age, sex, and race, equations that combine creatinine and CysC can provide accurate eGFRcr-cys predictions [73]. Other studies show that eGFRcys is not more accurate than eGFRcr, but eGFRcr-cys is more accurate than either alone [72,74,75]. Another factor allowing for greater individual accuracy is indexing GFR to certain body parameters; however, there are cases where non-indexed GFR might be preferred; for example, for drug dosing when there is a great difference between the non-indexed and indexed values or when there is a large change in body surface area after bariatric surgery [76].

When estimating GFR in patients with CKD, it is crucial to select formulas that minimize bias from body composition variations, particularly when assessing patients with obesity or sarcopenia. The most commonly used formulas for GFR estimation, such as the CKD-EPI (Chronic Kidney Disease Epidemiology Collaboration) equation, do not include body weight as a variable. Instead, they rely on serum creatinine, age, sex, and race to provide an estimate of kidney function. This approach helps avoid the inaccuracies introduced by body weight.

For patients with significant deviations in body composition, particularly those with low muscle mass (sarcopenia) or obesity, additional measures such as the CKD-EPI CysC or combined CKD-EPI creatinine-CysC equations are recommended, as they reduce the impact of muscle mass on serum creatinine levels. These equations are preferred in cases where more precise estimations of GFR are required, and they align with current guidelines advocating for non-weight-based methods for GFR calculation.

## 6. Evaluating Kidney Function in Obesity

The practice of indexing GFR to the body surface aims to equalize the level of metabolic waste exposure among people of various body sizes [77]. It is important to note that indexing GFR to body surface in obese individuals leads to an underestimation of the true underlying GFR whereas indexing GFR for body height or lean body mass leads to a better estimation of GFR [78]. As such, measurements of absolute GFR without indexing to the body surface are recommended in this population [79]. Additionally, the endogenous biomarkers typically used to estimate GFR can introduce bias. Creatinine levels are affected by muscle mass, while CysC is associated with fat mass [77]. Obesity is associated with a significant decrease in lean mass, given that the main factor of creatinine production is skeletal muscle mass. Equations that estimate GFR based on serum creatinine, such as the Cockcroft–Gault Equation, MDRD (Modification of Diet in Renal Disease), and CKD-EPI significantly underestimate GFR in obese patients. It is worth noting that some creatinine-based equations have been validated in obese populations, including the Salazar–Corcoran equation, the Mayo Clinic Quadratic equation (MCQ), and CKD-EPI [66]. CysC as a glomerular filtration marker, even though it is resistant to alterations caused by muscle mass variation, may be biased in people suffering from obesity or in those who have large variations in fat mass levels over time like, following bariatric surgery [80]. This is because of the association between fat mass index and higher CysC levels in the obese population, with eGFRcys particularly demonstrating the strongest association between fat mass index and kidney function, in contrast to eGFRcr-cys and eGFRcr [81]. CysC synthesis was found to be upregulated in adipose tissue, which may be the reason why higher BMI levels were independently associated with high CysC levels [74]. A higher CysC level is also independently associated with other factors such as older age, male gender, greater weight, greater height, current cigarette smoking, and higher serum CRP levels [82]. Many factors influencing these endogenous markers may render GFR estimation suboptimal for obese individuals and necessitate a wider availability of exogenous filtration markers as well as continued research to identify other endogenous markers that fit all the criteria and are resistant to unwanted influences.

At present, the most accurate method to estimate GFR in obese patients is to calculate a non-indexed eGFR by averaging the creatinine- and CysC-based equations. This conclusion is based on comparisons of indexed and non-indexed eGFR with mGFR obtained using iohexol clearance [66]. The most reliable way to evaluate GFR in severely obese individuals remains to measure it using a non-creatinine-based method, using exogenous filtration markers, and expressing it without indexing [66,79]. If eGFR should be obtained using a creatinine-based equation, such as MDRD or CKD-EPI, this should be performed by de-indexing it, dividing body surface area by 1.73 m^2^ (Table 1). In cases where only classic creatinine-based equations are available, using the patient’s lean weight instead of theoretical or actual weight in the Cockcroft–Gault equation, for example, can partially reduce biases (Table 1) [66,79]. Another study compared the accuracy of different formulas for estimating GFR in forty-five obese patients (grade I obesity: 48.89%). Here, the equations are indexed with ideal body weight as opposed to body surface area or lean weight. For grade I obesity, the Salazar ideal body weight formula showed the highest accuracy (% error = 2.30 ± 33.92), followed closely by the Cockcroft–Gault ideal body weight (% error = −2.84 ± 32.76). In patients with grade II and III obesity, the CKD-EPI formula had the best accuracy (% error = 3.84 ± 41.79), followed by the MDRD formula (% error = 4 ± 38.43). It was concluded that the substitution of ideal body weight for calculated body surface area improved GFR estimation accuracy [83].

## 7. Prognostic Equations and Risk Prediction

The availability of biomarkers and prognostic equations is crucial for predicting adverse kidney and cardiovascular outcomes in patients with obesity. Kidney function measures, eGFR, and albuminuria are essential in individual risk estimation and are strong prognostic biomarkers. An eGFR reduction below 60 mL/min/1.73 m^2^ or simply a small increase in albuminuria levels is associated with a markedly increased risk for cardiovascular events, all-cause mortality, and ESKD, both in those with already established CKD and the general population [84,85,86]. Markers of oxidative stress, tissue remodeling, and metabolism—myeloperoxidase, matrix metalloproteinases, tissue inhibitor of metalloproteinases-1, and fibroblast growth factor-23—are linked to atherosclerosis, cardiovascular disease, and a decline in eGFR in CKD [87,88,89]. Cardiac biomarkers and natriuretic peptides reflect the presence of cardiac and kidney dysfunction, complicating their use in individual risk prediction. Further research is needed to clarify the role of cardiac biomarkers in cardiovascular and renal risk prediction [86,90]. Proteomics, metabolomics, and genomics have introduced new biomarkers for CKD. “Omics” techniques are used to classify patients into distinct clinical or risk categories using a combination of informative peptides and metabolites. Tools like CKD273, a panel of 273 urine peptides, predict eGFR decline and reclassify patients for CKD progression risk [91]. A panel of 13 urine metabolites, including 3-HIBA and aconitic acid, was linked to CKD progression [92]. Genetic factors also play a role, with single-nucleotide polymorphisms like those in the UMOD gene and APOL1 variants associated with increased CKD risk and progression. Genetic risk scores combining single-nucleotide polymorphism data can predict eGFR decline and kidney outcomes. Studies from the UK Biobank further contributed to understanding CKD risks, highlighting the role of testosterone and fasting insulin [86,93]. Markers of tubule injury and tubule dysfunction also serve as indicators of acute kidney injury as well as CKD progression and cardiovascular disease [86].

Obesity and adiposity are predictive risk factors for the development of CKD. In a meta-analysis encompassing 3,504,303 patients, 521,216 of which were obese, and with an average follow-up period of 9.86 years, the relative risk of developing CKD in obese individuals was 1.81 (95% CI: 1.52–2.16) according to the random effects model. Obese individuals have a 1.81 times higher risk of developing CKD compared to the non-obese population, making obesity a crucial risk factor in preventive healthcare measures [14]. Perirenal fat thickness correlates with significant metabolic risk factors that affect kidney function, establishing a link between obesity and the progression of kidney disease. Perirenal fat thickness is greater in CKD patients with impaired fasting glucose compared to those with normal glucose levels (1.10 ± 0.40 cm vs. 0.85 ± 0.39 cm, *p* < 0.01), with patients in CKD stages 4 and 5 ([GFR] < 30 mL/min/1.73 m^2^) having the highest perirenal fat thickness. Serum triglyceride levels correlated positively with the perirenal fat thickness. In patients with a GFR < 60 mL/min/1.73 m^2^, uric acid levels correlated positively with the perirenal fat thickness (*p* < 0.05) [94].

A widely referenced risk prediction equation is the kidney failure risk equation (KFRE); however, it has gained significant criticism since its publication in 2011. Initially derived and externally validated in two Canadian CKD cohorts, concerns about its generalizability arose. A large international validation study involving 721,357 participants from 31 populations and CKD cohorts demonstrated excellent discrimination by the KFRE, though non-North American cohorts required a calibration factor [95]. A key criticism is that the KFRE estimates risk using predictor eGFR values from a single point in time without considering their longitudinal trajectory. Despite this, a study comparing a dynamic measurement with the original KFRE found only a slight improvement in predictive performance when accounting for changes over time [96]. Furthermore, the KFRE was developed using Cox or proportional hazard regression, a method used in survival analysis to explore the relationship between the survival time of subjects and several predictor variables, that did not account for the competing risk of death. This can lead to an overestimation of kidney failure risk, particularly among older CKD patients. The use of creatinine-based eGFR as discussed previously may lead to inaccuracies in predicted kidney failure risk in sarcopenic elderly patients or with race differences [97,98].

## 8. Mechanisms of the Kidney Effects of Obesity

Lipid metabolism and transport in kidneys

CD36 is the main mediator of fatty acid uptake in kidneys, and its expression increases in kidney damage [99]. Apart from CD36, several fatty acid transport proteins and fatty acid binding proteins modulate lipid synthesis, transport, and storage in kidney cells as well as interact with various nuclear transcription factors [100,101,102]. Lipid accumulation may result in inflammation, reactive oxygen species production, and endogenous electrical stress generation in renal cells, causing glomerular and tubulointerstitial damage [103,104]. In a study on proximal epithelial cells, oxidized HDL cholesterol is shown to induce apoptosis, reactive oxygen species production, and pro-inflammatory pathways via CD36 [105].

Obesity, increased inflammation, and kidney damage

Many studies suggest that individuals with obesity have a greater likelihood of developing CKD and progressing to ESKD [106,107]. Many interrelated mechanisms are suggested to play a role in the development of CKD in these patients. First of all, increased cytokine production by adipocytes and infiltrating macrophages such as TNF-α and IL-6 is associated with increased insulin resistance and kidney damage in obese individuals [26]. In addition to TNF-α and IL-6, leptin and adiponectin are among the adipokines produced by adipocytes and take part in CKD progression. Leptin levels increase in obese patients, and it stimulates mesangial and glomerular hypertrophy, resulting in glomerular sclerosis by inducing the expression of profibrotic genes and pro-inflammatory cytokines such as TGF-β1 [108]. Adiponectin levels decrease with increasing body weight, and decreased levels of adiponectin are associated with increased proteinuria and GFR since adiponectin has insulin-sensitizing, anti-inflammatory, and anti-apoptotic properties [109,110].

Obesity, AH, and kidney damage

Animal experiments show that weight gain results in increased blood pressure. This is also supported by clinical studies showing decreased blood pressure after weight loss in obese individuals. Evidence from these studies suggests that renal injury plays a significant role in the development of AH, and AH further exacerbates renal injury in obese patients [111,112]. Increased caloric intake and weight gain first result in increased renal blood flow and GFR. This phase of glomerular hyperfiltration is transient, as increased glomerular wall tension and hypertrophy lead to subsequent nephron loss, which causes GFR to decrease eventually [113,114]. In line with these mechanisms, focal segmental glomerulosclerosis and glomerulomegaly are the most common renal lesions in obese patients [115]. Obese individuals may consequently develop nephrotic-range proteinuria even in the absence of other comorbidities such as DM and AH [116]. Different mechanisms play a role in this process, as explained below. Obese individuals have increased plasma norepinephrine and urinary norepinephrine excretion, suggesting increased sympathetic nervous system activity [111,117]. Increased leptin in these individuals may be contributing to the activation of the sympathetic nervous system, as leptin infusion increases sympathetic nervous system activity and blood pressure regardless of food intake and body mass in animal studies [118,119]. Increased sympathetic nervous system activity is suggested to control increased sodium reabsorption via renal nerves, as renal denervation abolishes these effects in animal studies [120].

Furthermore, many clinical observations and animal studies identified increased RAAS activation in obesity [121,122]. Obesity increases sensitivity to angiotensin II via mechanisms that are still not clear. Angiotensin II acts on different NaCl transporters to stimulate sodium reabsorption and induce efferent arteriole constriction, which further increases glomerular hydrostatic pressure [123]. Treatment with ACE-I decreased ESKD risk in obese patients in a clinical trial, which is in line with these mechanisms [124]. Aldosterone increase is suggested to play a role in the development of resistant AH in obese individuals, as spironolactone treatment results in significantly reduced blood pressure and proteinuria in these patients [125]. In animal studies, mineralocorticoid receptor antagonism reduced blood pressure and sodium retention despite increased activation of RAAS, suggesting a considerable role for this mechanism, which needs further confirmation in humans [126]. In addition, increased perirenal and renal sinus fat in obesity compresses kidneys. Renal extracellular matrix expansion in obesity also takes part in this compression, as shown in animal studies [127,128]. Visceral fat accumulation in obesity increases intraabdominal pressure, further compressing the kidneys. Increased pressure in and around the kidneys compresses the loop of Henle, contributing to increased sodium reabsorption.

All in all, the activation of the sympathetic nervous system and RAAS as well as renal physical compression impair renal pressure natriuresis and result in increased reabsorption of sodium and expanded extracellular fluid volume. Reduced sodium delivery to macula densa results in the alteration of the tubuloglomerular feedback mechanism, resulting in compensatory renal afferent arteriole vasodilation, increased renal blood flow, and GFR, resulting in the changes explained above. As a result, obese individuals maintain their sodium balance at higher blood pressure compared to their lean counterparts, and these increased blood pressures further damage the kidneys [129].

Obesity, microalbuminuria, and renal damage

The relationship between obesity and microalbuminuria is crucial to elucidating the early stages of CKD. A cross-sectional study involving a total of 41,085 patients without any known kidney diseases illustrated that patients with both central and peripheral obesity are at increased risk for elevated urinary albumin-to-creatinine ratio (UACR) (OR: 1.14, 95% CI: 1.07 to 1.12, *p* < 0.001) after adjustment for multiple confounding factors [130]. Similarly, obesity-related parameters, including high body-mass index, waist-to-hip ratio, and waist-to-height ratio, are all significantly associated with both albuminuria and lower eGFR among type II DM patients [131]. Similar patterns have been reported in other population-based clinical studies as well [132,133]. However, the validity of the UACR among obese or overweight individuals is unclear, as the urinary creatinine excretion is a reflection of body creatinine generation primarily occurring at skeletal muscle tissue [134]. Body composition studies have identified variable outcomes in terms of lean body mass or skeletal muscle mass among obese or overweight individuals, as obesity is not a uniform condition with multiple phenotypes, including sarcopenic obesity [135,136]. Few approaches aiming to adjust for variabilities in muscle mass and obesity for UACR have been postulated with currently limited clinical use [137]. Nevertheless, albuminuria is a widely available clinical assessment tool to evaluate kidney damage among overweight and obese patients. However, there is currently no clinical tool or cutoff value for albuminuria or proteinuria to guide decision-making for kidney biopsy.

Obesity, insulin resistance, and renal damage

Type 2 DM is an important complication of obesity. Increased inflammation, hyperinsulinemia, and mitochondrial dysfunction encountered in obesity predispose obese individuals to develop insulin resistance in target organs (muscles, liver, adipose tissue), which results in the occurrence of type 2 DM [138]. Type 2 DM provokes renal damage via various mechanisms, which can be discussed under the term diabetic kidney disease. Hyperglycemia can provoke glomerular hyperfiltration due to increased glucagon concentrations, which provoke the vasodilation of afferent arterioles [139]. Furthermore, hyperglycemia can induce RAAS activation and AH, which provoke the vasoconstriction of efferent arterioles, further increasing the glomerular pressure. As in the case of AH explained above, increased glomerular pressure causes subsequent renal injury [140]. Hyperglycemia itself can also provoke inflammation and fibrosis in kidneys by activating protein kinase C and JAK-STAT pathways and by generating advanced glycation end products, reactive oxygen species [141].

One should also note that CKD itself is an important risk factor for the development of insulin resistance. Rat adipocyte tissues incubated in serum taken from CKD patients show decreased glucose uptake [142]. Studies in CKD patients show a decreased glucose uptake in skeletal muscle despite normal gluconeogenesis and glucose uptake in the liver [143,144]. Decreased physical activity, vitamin deficiency, metabolic acidosis, oxidative stress, and deranged adipokines in CKD patients are all suggested to play a role in the development and progression of insulin resistance [145]. All in all, obesity provokes insulin resistance and subsequent renal damage, and the resulting renal damage further makes it difficult to regulate already deranged blood glucose in these patients.

Obesity, non-alcoholic fatty liver disease (NAFLD), and renal damage

**Figure 2:**
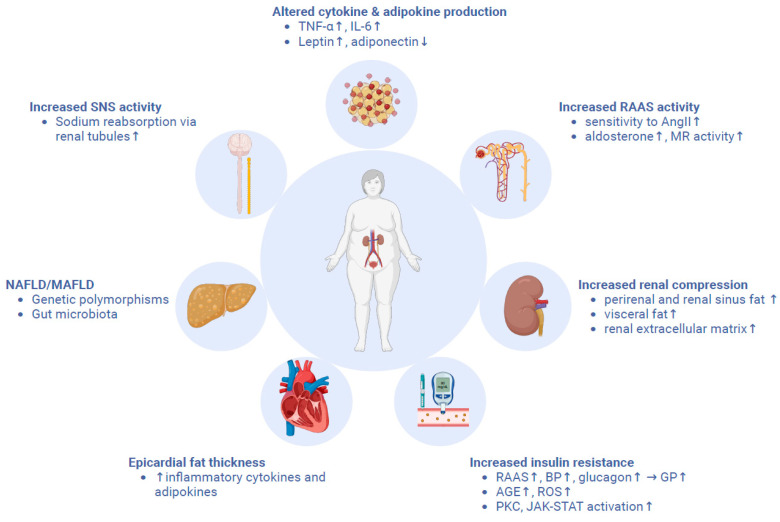
The pathophysiological mechanisms linking obesity and CKD.

NAFLD is the most frequent liver disease seen worldwide and is present in almost all moderately to severely obese patients [146]. In fact, obesity is known to play an important role in the development of NAFLD via various mechanisms, which are not completely understood to date. Most importantly, some adipokines secreted by the adipose tissue influence RAAS and podocyte viability in addition to controlling hepatic fibrosis in some animal models [147]. Insulin resistance, as explained above, also takes part in the occurrence of NAFLD in obese patients. Being a multisystemic disease, NAFLD is associated with an increased risk of developing KD, regardless of the presence of established CKD risk factors [148,149]. For its multisystemic associations, some experts in the field propose “metabolic associated fatty liver disease” (MAFLD) as a new term instead of NAFLD, and the diagnosis requires at least one metabolic criterion in addition to the evidence of hepatic steatosis [150]. The mechanisms linking MAFLD or NAFLD and CKD are not yet well understood, but both genetics and environmental factors are thought to take part in the process. Genetic polymorphisms in some genes, such as PNPLA3, HSD17B13, TM6SF2, MBOAT7, and GCKR, are associated with both NAFLD and CKD pathogenesis; they are thus thought to be monitoring the interplay between these two diseases [151]. Furthermore, recent studies show that gut microbiota may also be taking part in this interplay via the “gut-liver-kidney signaling axis” [152]. In fact, some of the metabolites produced by gut microbiota, such as indoxyl sulfate, p-cresyl sulfate, and trimethylamine N-oxide, are toxic and are eliminated by the kidney. The decreased levels of short-chain fatty acids promote increased production of these toxins in the context of CKD, and the elevated plasma levels of these toxins can have adverse effects on both the kidney and the liver [153]. The hypothetical pathophysiological mechanisms linking CKD and obesity are summarized in Figure 2.

## 9. Therapeutic Approaches

**Figure 3:**
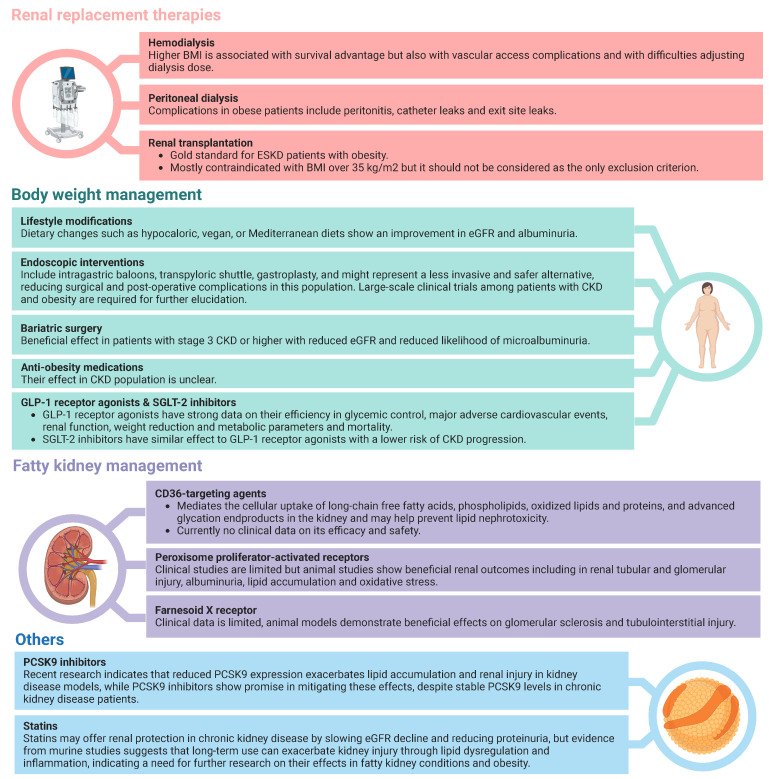
Therapeutic approaches towards the management of kidney function and obesity among CKD patients with obesity.

Potential therapeutic options for the management of CKD and obesity are summarized in Figure 3.

Body Weight Management Strategies

The management of obesity among CKD patients is a challenging topic, though a crucial area of interest as high BMI is associated with poor kidney outcomes. The diagnosis of CKD is mostly based upon eGFR and albuminuria, while assessment of both parameters is challenging among patients with a higher BMI. Spot UACR mostly replaces the gold standard assessment method of proteinuria, namely 24 h urine collection, which may underestimate proteinuria among obese or overweight patients as the nominator spot urine creatinine is proportional to muscle mass, which is commonly higher among those subjects [137]. Potential approaches to overcoming such bias are the utilization of different cutoff values for proteinuria definition in obese patients for which there is no established cutoff and the utilization of various models for creatinine excretion estimation with three available models [154,155,156]. Similarly, serum creatinine-based estimations for GFR have considerable limitations as they are based upon body surface area as well, despite being commonly utilized [78]. Despite such bias in the measurement of GFR and albuminuria in obese or overweight patients, there are different diagnostic criteria for CKD for those individuals. Also, the follow-up of those patient populations is similar to the general population. Lifestyle modifications, including exercise models and dietary interventions, bariatric surgery, and GLP-1 analogs, have been shown to be beneficial in obese CKD patients in large-scale randomized clinical trials [2,157,158].

Lifestyle modifications

Multiple dietary strategies have been evaluated in CKD patients with higher BMI with variable success. Hypocaloric diet has been associated with a statistically significant decline in body weight accompanied by improvement in albuminuria in prospective clinical trials, while most of those trials have been conducted on either diabetic kidney disease or proteinuric nephropathy patients [116,159,160]. Similarly, a vegan diet or diet rich in vegetables and fruits has been investigated in CKD patients and has been linked to a considerable decline in albuminuria [161,162]. A randomized control trial involving a total of 318 CKD stage I–III patients over a two-year clinical trial period has demonstrated beneficial effects of low-carbohydrate (+5.3% [95% CI 2.1–8.5]), Mediterranean (+5.2% [3.0–7.4]), or low-fat (+4.0% [0.9–7.1]) diets on eGFR with similar magnitude. A similar pattern of improvement in microalbuminuria has been reported in all dietary intervention groups (*p* < 0.05) [163]. Similarly, the beneficial effects of exercise on renal function among obese or overweight CKD patients have been demonstrated in clinical trials and a large-scale meta-analysis study [164,165,166]. Therefore, dietary interventions along with exercise programs should be offered to all CKD patients with BMI at or over 30 kg/m^2^ unless contraindicated for any reason.

Endoscopic interventions

Bariatric endoscopic procedures, either as primary treatment or revisional procedure following surgical approach failure, have emerged as a less invasive therapeutic approach for the management of obesity and associated complications [167]. Potential bariatric endoscopic procedures are classified as restrictive procedures, including intragastric balloons, transpyloric shuttles, gastroplasties, or malabsorptive procedures such as duodenal mucosal resurfacing or bypass operations [168]. Large-scale meta-analysis studies have revealed the efficiency and safety of bariatric endoscopic procedures, though such procedures are mostly associated with a lower degree of total body weight loss and complications compared to bariatric surgery [169,170,171]. Even though such therapeutic options appear to be valid for CKD patients, the literature regarding their use among CKD or ESKD patients is highly limited. Endoscopic intragastric balloon placement appears to be a safe method that leads to moderate weight loss [172,173], though few case reports have linked such a therapeutic modality to acute kidney injury [173,174]. Also, an endoscopic sleeve gastroplasty procedure has been successfully implemented in an ESKD patient undergoing peritoneal dialysis [175]. Even though incretin-based therapies are known to lead to gastroparesis and gastrointestinal adverse events that may be further exacerbated by endoscopic procedures, our knowledge regarding the combination of endoscopic procedures with pharmacotherapeutic approaches is highly limited. Endoscopic therapeutic options appear to be safe and efficient management options for CKD patients with obesity, as such patients are more prone to developing higher surgical and postoperative complication rates; nevertheless, there is a clear need for large-scale clinical trials evaluating such therapeutic options among CKD patients for a better understanding of this issue.

Bariatric surgery

Multiple bariatric surgical interventions have been studied among obese CKD patients in terms of efficiency, kidney and cardiovascular outcomes, and complications. Such surgical intervention methods include sleeve gastrectomy, Roux-en-Y gastric bypass, laparoscopic adjustable gastric band, and vertical banded gastroplasty. All of those modalities have been implemented with variable success among CKD patients [176,177,178,179]. A meta-analysis study involving a total of 19 clinical trials has demonstrated beneficial effects of bariatric surgery in patients with stage III or above CKD on eGFR (MD: 11.64 (95% CI: 5.84 to 17.45, I2 = 66%) mL/min/1.73 m^2^) and reduced likelihood for microalbuminuria (RR of −3.03 (95% CI: −1.44 to −6.40, I^2^ = 91%) [180]. A similar pattern of improvement in renal functions, including albuminuria and eGFR, has been reported in another meta-analysis study involving a total of 8515 patients from 49 clinical studies [181]. To conclude, bariatric surgery has led to beneficial outcomes in terms of renal function among CKD patients and, thus, should be offered to patients meeting the criteria for bariatric surgery.

Anti-obesity medications

There are a few Food and Drug Administration (FDA)-approved pharmacotherapeutic approaches for obesity management, including orlistat, phentermine, phentermine-topiramate combination therapy, liraglutide, and bupropion-naltrexone combination therapy. Nevertheless, the use of those medications among the CKD population is unclear, with limited data regarding dose adjustments and safety. Only liraglutide appears to be safe and efficient in all CKD stages, while the utilization of the rest of the medications is mostly not recommended.

GLP and GIP-1 receptor agonists

GLP-1 receptor agonists are a novel anti-diabetic medication class with strong data regarding their safety and efficiency in terms of glycemic control, major adverse cardiovascular events, renal functions including eGFR and albuminuria, weight reduction and metabolic parameters, and mortality in large-scale randomized clinical trials and meta-analysis studies [182]. The potential nephroprotective mechanisms of GLP-1 receptor agonists include reduction in oxidative stress and pro-inflammatory cytokines via inhibition of protein kinase A and C and NF-κB signaling pathways, reduction in glomerular AH, and regulation of indirect nephrotoxic mediators such as hyperglycemia, AH, obesity, and dyslipidemia [183]. Multiple randomized clinical trials have investigated the renal effects of GLP-1 receptor agonists along with other clinical parameters, while major clinical trials in this field include ELIXA (lixisenatide), LEADER (liraglutide), SUSTAIN-6 (semaglutide), EXSCEL (exenatide), REWIND (dulaglutide), and AMPLITUDE-O (efpeglenatide) trials [184]. A meta-analysis study evaluating those clinical trials has demonstrated an approximately 21% risk reduction for composite kidney outcome (HR 0.79 [95% CI 0.73–0.87]; *p* < 0.0001) while the composite renal end-point was reached in all trials except for the ELIXA and EXSCEL trials [182]. Similar beneficial effects have been validated from real-world data based on a Scandinavian cohort study [185] and a United States veterans cohort study [186]. A meta-analysis study conducted in 2023 over a total of 75.346 patients from multiple cohorts has demonstrated similar renal outcomes with GLP-1 receptor agonist therapy, including eGFR and albuminuria, while treatment with dual GIP/GLP-1 receptor agonist tirzepatide therapy has led to superior renal outcomes, including 45% risk reduction for kidney-specific composite outcome (HR 0.55, 95% CI 0.40–0.77) and 62% risk reduction for worsening albuminuria (HR 0.38, 95% CI 0.24–0.61) [187,188].

Management Options for Fatty Kidney

A novel concept of “fatty kidney” has emerged recently referring to the effects of ectopic or renal sinus fat accumulation that may potentially progress to CKD, as in metabolically associated fatty liver disease leading to cirrhosis, through various pathophysiological mechanisms including inflammation, oxidative stress, glomerular and tubular injury, and endothelial dysfunction [189]. Although weight reduction strategies, including lifestyle interventions, and pharmacotherapeutic options, including RAAS blockers and SGLT-2 inhibitors, have been shown to be beneficial in the management of fatty kidney, multiple other hypothetical therapeutic approaches exist for such a condition as well.

CD36-targeting agents

CD36, also known as scavenger receptor-B2, is a receptor mediating the cellular uptake of long-chain free fatty acids, phospholipids, oxidized lipids and proteins, and advanced glycation end-products located mostly at renal proximal and distal tubular epithelium and podocytes with considerable upregulation in CKD settings [190]. The lipid nephrotoxicity hypothesis, first proposed by Moorhead and colleagues in 1982, refers to the association between dyslipidemia and the progression of CKD [103]. The uptake of oxidized lipids and advanced glycation end-products by CD36 into proximal tubular cells has led to the activation of the NLRP3 inflammasome and therefore the secretion of pro-inflammatory cytokines, including interleukin-1 and tumor necrosis factor-alpha. Moreover, the CD36-mediated signaling pathway induces protein kinase-C, leading to the activation of the NF-kB pathway and the production of reactive oxygen species and certain pathways triggering apoptotic signaling routes. The end result of overexpressed CD36 is the activation of pro-inflammatory, pro-fibrotic, and apoptotic signaling pathways and the formation of reactive oxygen species, all of which result in CKD progression [190]. Therefore, hypothetically, CD36-targeting therapies may reverse or prevent the progression of CKD, especially in cases with dyslipidemia and obesity. A pre-clinical study conducted on mice has demonstrated prevention of CKD progression in mouse models with either CD36-knockout or when treated with CD36 antagonist, namely 5A peptide [191]. Another cell line study has demonstrated inhibition of CD36-mediated activation of ERK-1/2 and Smad-2 pathways, generation of reactive oxygen species, and epithelial-to-mesenchymal transition at tubular epithelium when treated with a CD36 antagonist, namely sulfosuccinimidyl-oleate, under a high glucose environment [192]. However, there is currently no clinical data regarding the efficiency or safety of CD36-targeting therapies.

Farnesoid X receptor (FXR)

FXR is a bile acid receptor primarily expressed in the liver and intestines and recently identified in renal tissue, with activation associated with inhibition of pro-inflammatory and pro-fibrotic signaling cascades and reduction in oxidative stress [193]. Although clinical data regarding the use of bile acid sequestrants targeting FXR in terms of renal function is highly limited, pre-clinical data from animal models have demonstrated beneficiary effects of obeticholic acid therapy on histopathological features including glomerular sclerosis and tubulointerstitial injury [194,195]. Despite the lack of efficacy data on humans regarding renal functions, the safety of bile acid sequestrants is well-established during their use in hyperlipidemia and liver diseases.

There are a few other hypothetical target molecules that may have clinical and therapeutic significance in the management of CKD among obese or overweight patients: (1) Sterol regulatory element binding protein (SREBP)-1 is a transcription factor functioning in the production and cellular transport of fatty acids and cholesterol molecules and over-expressed in high-fat diet receiving animal models that may be reversed with insulin therapy, leading to a decline in renal fat accumulation, oxidative stress, and inflammation [196,197,198]; (2) the Janus-activated kinase/signal transducer and activator of transcription (JAK-STAT) pathway, which is over-expressed in CKD patients [199]; (3) C/EBPα and C/EBPβ, two transcription factors regulating the expression of proteins involved in lipogenesis [104]. However, there is a lack of clinical data regarding such therapeutic targets in terms of efficacy or safety, with a definitive need for future large-scale clinical trials.

Others
  Proprotein Convertase Subtilisin/Kexin Type 9 (PCSK9) inhibitors

PCSK9 inhibitors lower LDL cholesterol by blocking the PCSK9 protein, downregulating hepatic LDL receptor expression, and causing higher plasma LDL cholesterol [200]. By preventing LDL receptor degradation, PCSK9 inhibitors enhance LDL clearance from the bloodstream [200]. PCSK9 expression is significantly reduced in the glomeruli and podocytes of diabetic kidney disease models, leading to increased lipid accumulation in kidney tissues [201]. In PCSK9 knockout mice, there was a notable rise in lipid levels, mitochondrial injury, and podocyte apoptosis compared to wild-type counterparts, indicating that reduced PCSK9 exacerbates renal injury through lipid dysregulation and mitochondrial dysfunction [201]. In another study, mice lacking PCSK9 showed increased CD36 expression, leading to heightened lipid accumulation, ER stress, inflammation, and renal damage [202]. Conversely, treatment with the anti-PCSK9 monoclonal antibody evolocumab reduced CD36 levels and protected against HFD-induced renal lipotoxicity [202]. The PCSK9Qβ-003 vaccine significantly reduced cholesterol levels and improved renal function in mice with hypercholesterolemia and renal fibrosis by enhancing fatty acid β-oxidation and reducing renal lipid accumulation and fibrosis, indicating its potential as a therapeutic approach for these conditions [203]. In nondiabetic patients with CKD and not on statins, plasma PCSK9 levels were not significantly linked to eGFR or proteinuria across CKD stages and remained stable among these stages [204], indicating that kidney function may not impact PCSK9 metabolism. Similar results were shown by another study with a nondialysis CKD cohort [205].

Statins

Statins, lipid-lowering agents, appear to offer renal protection through both cholesterol reduction and non-cholesterol-mediated mechanisms; they have been associated with a deceleration in the decline of GFR and a reduction in proteinuria in the CKD population [206]. Although not specifically focused on fatty kidney conditions or patients with obesity, a systematic review and meta-analysis of 57 randomized controlled trials involving 143,888 non-dialysis participants with kidney disease found that statin therapy did not significantly reduce the risk of kidney failure events or ESKD [207]. However, it was associated with a modest reduction in proteinuria and a slower rate of decline in the eGFR [207]. Additionally, statin treatment significantly lowered the risk of cardiovascular events in patients with CKD [207,208]. Concerning in vivo evidence, atorvastatin effectively mitigates kidney injury and restores renal organic anion transporter 3 functions in high-fat diet-induced obese rats by reducing oxidative stress, inflammation, and renal lipid accumulation [209]. Long-term statin administration in db/db mice worsens renal injury, characterized by kidney atrophy, glomerular hypertrophy, mesangial expansion, thickened basement membranes, increased podocyte loss, and elevated urinary KIM-1 and serum creatinine levels [210]. The treatment exacerbates renal fibrosis and inflammation, indicated by heightened macrophage infiltration and elevated IL-1β expression. At the same time, RNA sequencing reveals dysregulation of lipid metabolism that leads to increased lipid droplet accumulation in the kidneys and reduced fatty acid oxidation [210]. Despite lowering serum lipid levels, statins upregulate lipid uptake mechanisms in the kidneys, contributing to renal injury [210]. Overall, evidence indicates that statins can benefit patients with CKD by slowing kidney function decline and reducing adverse events. However, research on fatty kidney and obesity-related complications is largely limited to a few murine studies, highlighting the need for further investigation to clarify their specific effects.

## 10. Conclusions

In conclusion, obesity presents a multifaceted challenge in the context of CKD, influencing both its development and progression through diverse mechanisms. Mutually, CKD poses a distinct challenge for obesity, affecting its management, diagnosis, and prognostic implications. This review also underscores the significant association between elevated BMI and the increased risk of advanced CKD stages, highlighting the nuanced impact of different obesity phenotypes such as sarcopenic obesity and metabolically healthy versus unhealthy obesity. Meanwhile, assessing kidney function in obese individuals presents unique challenges due to the limitations of traditional markers like creatinine and CysC, which are influenced by muscle and fat mass variations. Given these complexities, current recommendations lean towards using non-indexed equations and exploring alternative biomarkers for more accurate estimations in this population.

While lifestyle changes like hypocaloric diets and exercise programs should be a cornerstone of treatment, bariatric surgery and endoscopic procedures offer promising alternatives for patients with severe obesity who fail conservative approaches. The advent of GLP-1 receptor agonists, particularly when combined with GIP, presents an exciting therapeutic avenue, showing not only weight reduction but also significant nephroprotective effects. Emerging concepts like ‘fatty kidney’ highlight the need for targeted therapies such as CD36 inhibitors and FXR activators, though much of this remains in pre-clinical stages. Despite the progress, the safety, efficacy, and combination of various treatments—pharmacotherapies and surgical approaches—remain limited and require further investigation through large-scale clinical trials.

Future obesity research should concentrate on several pivotal areas to enhance understanding of tailored treatment strategies and therapeutic efficacy. Primarily, there is a critical need to establish an optimal formula for estimating GFR that accommodates diverse populations, including obese individuals, necessitating investigations to prevent confounding results in obesity and CKD research. Furthermore, elucidating the distinct pathophysiological mechanisms underlying different obesity phenotypes is essential to transitioning from theoretical models to comprehensive clinical insights. The formulation of evidence-based treatment guidelines is imperative, given the experimental nature of current obesity interventions; thus, research should focus on developing standardized protocols that incorporate personalized medicine. Additionally, large-scale population studies stratifying participants by phenotypic and metabolic obesity types will yield robust data to inform individualized treatment strategies and elucidate the robust statistical significance of this categorization for forecasting prognosis. Addressing these areas will substantially advance the understanding of obesity and optimize management strategies, ultimately improving patient outcomes.

## Figures and Tables

**Table 1:** Creatinine-based and combined creatinine-CysC equations glomerular filtration rate equations validated in obese populations.

Creatinine Equations	Equation Formula
Cockcroft–Gault Equation (Lean Weight)	CrCl = (140-age × lean body mass)/SCr × 72
Mayo Clinic Quadratic Equation (MCQ) usingonly creatinine	eGFR = exp (1.911 + 5.249/SCr [mg/dL] − 2.114/SCr [mg/dL] − 0.00686 × age (years) (−0.205 if female)
Salazar–Corcoran Equation	CrCl = [137-age] × [0.285 × weight] + (12.1 × H)(51 × SCr) for males
	CrCl = [146-age] × [0.287 × weight] + (9.74 × H)(60 × SCr) for females
MDRD Deindexed (MDRDd)	eGFR = 175 × (Scr)^(−1.154)^ × (age)^(−0.203)^ × (0.742 if female) × (1.212 if black) × BSA of the patient/1.73
CKD-EPI Deindexed (CKD-EPId)	$\mathrm{eGFR}\;=141\;\times \;\min\;(\frac{Scr}{\kappa },1{)}^{\alpha }\;\times \;\max\;(\frac{Scr}{\kappa }$, 1)^(−1.209)^ × (0.993)^Age^ × (1.159 if black) × BSA of the patient/1.73 *
**Combined Creatinine-Cystatin C Equations**	**Equation Formula**
CKD-EPI Creatinine-Cystatin C Equation(CKD-EPI 2012)	eGFR = 135 × min(Scr/κ, 1)ᵅ × max(Scr/κ, 1)^(−0.601)^ × min(CysC/0.8, 1)^(−0.375)^ × max(CysC/0.8, 1)^(−0.711)^ × 0.995^n^ × (1.08 if female) × (1.159 if black) *
Mayo Clinic Quadratic Equation (MCQ)	eGFR = 79.901 × (CysC)^(−0.438)^ × (serum creatinine)^(−0.191)^ × (age^(−0.195)^) × (0.82 if female)
FAS (Full Age Spectrum) Equation	eGFR = 107.3 × (CysC/median CysC)^−0.4^ × (Scr/median Scr)^−0.2^
Larsson’s Formula	eGFR = 99.19 × (CysC)^(−1.713)^ × (creatinine)^(−0.451)^ × (0.996 if female) × (1.008 if age > 50)

* κ is 0.7 for females and 0.9 for males, α is −0.248 for females and −0.207 for males, and *n* is age in years. CrCl, creatinine clearance (mL/min); SCr, serum creatinine (mg/dL); eGFR, estimated glomerular filtration rate (mL/min/1.73 m^2^); H, height in meters; BMI, body mass index; BSA, body surface area; CysC, cystatin C concentration.
